# Ocular Toxoplasmosis among Livestock Farmers and Raw Meat Handlers in Uyo, Nigeria

**DOI:** 10.4314/ejhs.v31i2.8

**Published:** 2021-03

**Authors:** Emem Godwin Abraham, Anietie Effiong Moses, Uwemedimbuk Smart Olugbemi O Motilewa, Akaninyene Innocent Uwah, Eme Iwat Itina, Anthony N Umoh

**Affiliations:** 1 Department of Ophthalmology, University of Uyo Teaching Hospital, Uyo, Nigeria; 2 Department of Microbiology, University of Uyo Teaching Hospital, Uyo, Nigeria; 3 Department of Community Health, University of Uyo Teaching Hospital, Uyo, Nigeria

**Keywords:** Ocular toxoplasmosis, occupationally exposed, anti-T. gondiiIgG antibody

## Abstract

**Background:**

Toxoplasmosis is the commonest cause of infectious posterior uveitis in humans and can lead to blindness and low vision in both immune-competent and immunecompromised persons worldwide. The aim of this study was to determine the prevalence of Ocular Toxoplasmosis (OT) and potential risk factors among livestock farmers and raw meat handlers in Uyo.

**Methods:**

This was a descriptive cross-sectional community-based study involving clinical eye examination, laboratory detection of anti-Toxoplasma gondii IgG antibody and HIV testing. Participants' other information was obtained using interviewer-administered questionnaire.

**Results:**

There were 339 participants aged 15–78 (mean 34.8±11.6) years,283 (83.5%) were males 56(16.5%) were females; 189 (55.8%) tested seropositive for anti-Toxo. gondiiIgG antibodies. Eight (2.4%) had presumed ocular toxoplasmosis (POT); 6 of the 8 were seropositive for anti-T.gondiiIgG antibody; and 2 of the 8 POT (25%) were HIV-seropositive. Of the 189 who were anti-T.gondiiIgG antibody seropositive, 6 (3.2%) had OT. Factors associated with OT were age (31–50 years) and female gender (P = 0.049 and 0.001, respectively). HIV infection was associated with POT (P=0.033). Most of the ocular lesions (87.5%) were unilateral and located at the posterior pole (77.7%).

**Conclusion:**

The prevalence of presumed ocular toxoplasmosis (POT) and ocular toxoplasmosis (OT) among livestock farmers and raw meat handlers in Uyo are 2.4% and 1.8%, respectively. Potential risk factors are being female, and persons between fourth and fifth decades of life. Awareness creation on toxoplasmosis among this occupational group is advocated.

## Introduction

Toxoplasmosis is the commonest cause of infectious posterior uveitis in humans and can lead to blindness and low vision in both immune-competent and immune-compromised persons (1). Cats are definitive host of *Toxoplasma gondii* while humans and other mammals are intermediate hosts. Ocular toxoplasmosis often presents with unilateral chorioretinal lesion associated with vitritis (2). It was earlier presumed that adult ocular toxoplasmosis (OT) was a recurrence of congenitally acquired infection, but more recent reports indicate that acquired infections may account for larger portion of ocular involvement (3). Epidemiological report indicates that about a third of the world human population has been infected with *T. gondii*, making it one of the most important parasitic infections (1). Serological studies also estimate that 30–50% of the global population has been exposed and may be chronically infected although infection rates differ significantly by country (4). Toxoplasmosis is endemic in tropical Africa and South America, and most infections in Africa are acquired through improperly cooked contaminated meat, unwashed contaminated fruits, vegetables, meat chopping boards, unwashed contaminated hands, and by contact with infected cat droppings (5).

The seroprevalence of anti-*Toxoplasma gondii*IgG varies worldwide ranging from 14% in USA (6), 46% in Tanzania (7) to as high as 98% in Southern Brazil (8). Environmental conditions like eating habits, and hygiene as well as host susceptibility account for the global differences in the prevalence (7). In USA, toxoplasmosis is the second most common cause of deaths related to food-borne diseases, and one million new infections are being recorded each year, resulting in about 20,000 cases of retinal infection (9). In Nigeria, 28.7% seroprevalence of *T. gondii* infection has been reported in healthy adults and up to 54.2% prevalence among HIV-positive persons (10). In the University of Uyo Teaching Hospital, a prevalence of 0.28% of presumed ocular toxoplasmosis has been documented in routine eye patients seen at the eye clinic (11).

The asymptomatic nature of toxoplasmosis places a significant public health burden necessitating early laboratory detection and proper management of infected persons. Effect on the host depends on the strength of the host immune system. In immuno-competent individuals, infections often go unnoticed and often misdiagnosed as malaria or typhoid fever because of the low suspicion index amongst health practitioners (12). Ocular complication is more severe in immuno-compromised persons and second only to cytomegalovirus retinitis (1).

Toxoplasmosis is an occupationally hazardous disease among exposed persons. Many citizens of Akwa Ibom State and other contiguous States in South-South Nigeria are increasingly engaged in livestock farming notably cow, sheep, goat, poultry and piggery among others. Consumption of roasted pork and meat from different types of animals locally referred to as ‘suya’ is on the rise in the state. These ‘suya’ meal often times are not properly cooked and so if consumed may become a route of infection to unsuspecting consumers. Apart from livestock farmers, slaughter house workers and meat sellers are also at risk of acquiring *T. gondii* infection. Potential routes of transmission include skin abrasion or cut and aerosols or droplet exposure of organisms to the mucous membranes of the eye, nose, or mouth (13).

Akwa Ibom state currently has an HIV seroprevalence of 10.8% and has been consistently ranked among the five states with high HIV seroprevalence in Nigeria (14). The high HIV/AIDS prevalence in the state remains a potential risk determinant that could compound the severity of toxoplasmosis including ocular complications in immunecompromised persons. Therefore, this study aimed to investigate the prevalence of ocular toxoplasmosis (OT) in persons who were occupationally exposed to livestock and raw meat in Uyo.

## Materials and Methods

**Study area**: This study was conducted in three large public slaughter houses and animal markets in Uyo metropolis and its environs, namely; Ntak Inyang, Mbak Itam, and Akpan Andem livestock markets. Uyo is the capital city of Akwa Ibom state, Nigeria. Akwa Ibom State has a population of 3.9 million (15). The state is estimated to be growing at the rate of 3.4% annually with a projected total population of 5.5 million in 2016. It is ranked the 9th most populated state in Nigeria. Uyo is located along Latitude 5° 01′ 59″ N and Longitude 7° 55′ 35″ E with a projected population of about 805,451 and is one of the fastest growing cities in Nigeria, economically and population wise (15). It is also a commercial nerve center of Akwa Ibom State and can be accessed by inhabitants of surrounding Local Government Areas and neighboring states for economic and social activities. Subsistence agriculture (crop and animal rearing) accounts for 55% of the total workforce of the inhabitants.

**Study design**: This was a descriptive cross sectional community-based study where consented participants were tested for presence of anti-*Toxoplasma gondii*IgG antibody and anti-HIV antibody as well as examined clinically for eye complications. Questionnaires were administered by trained research assistants. Information collected included sociodemography, occupation and related issues, potential risk factors among others. Blood samples from all eligible participants were collected and screened in the laboratory for the presence of anti *T. gondii*IgG and HIV antibodies. Eyes of all participants were clinically examined for signs of ocular toxoplasmosis. The study was conducted between May 2016 and July 2017.

**Study population**: The study population included 339 male and female livestock farmers and slaughter house workers of all age groups present at the slaughter houses at the time of visit. Specifically, the participants comprised cattle, goat, sheep and chicken farmers/traders, butchers and meat sellers at the three large public abattoirs serving Uyo and environs. Persons of all age groups, diverse ethnic groups and educational status who actively participated in livestock farming and abattoir operations were included in the study. Livestock farmers/traders were those who raised livestock for sale at the abattoir/livestock markets. Butchers were those responsible for slaughtering the animals while meat vendors/sellers were those engaged in raw meat selling. There were also those who carried out bulk meat purchase at the slaughter houses and sold same to the retailers in the markets referred to as middlemen.

**Data collection**: Questionnaire was earlier pre-tested at a large slaughter house in Abak metropolis, Abak Local Government Area, a neighbouring town from the study site. Areas of ambiguity and questions that were unclear were adjusted accordingly for clarity before wide scale use of the instrument. Socio-demographic information of each of the participants was obtained at the time of sample collection through interviewer-administered questionnaires by well-trained research assistants. Information on potential risk-factors associated with toxoplasmosis was also collected in the formatted questionnaire.

**Sample collection**: Seven milliliters (7ml) of venous blood samples were aseptically collected from consented participants by qualified medical personnel using a vacutainer blood collection device and shared into plain and EDTA tubes. The blood samples were kept in ice-packed cooler and transported to the laboratory. Serum was separated from plain blood samples after centrifugation at 3000 rpm for 10 min and stored in 2 ml vials at -20 °C until required for testing.

**Sample analyses**: All universal precautions were strictly followed when carrying out laboratory analyses, which included assay for anti-*T. gondii*IgG antibody by ELISA technique and rapid HIV testing using the national testing algorithm.

**Serological testing of anti-Toxoplasma gondiiIgG antibody**: The determination of presence of anti-*Toxoplasma gondii*IgG specific antibody in serum of participants was carried out using the Enzyme Linked Immunosorbent Assay kit (ChemuxBioScience, Inc, California, USA) according to the manufacturer's manual. The optical density was measured in a spectrophotometer using a single wave length of 450 nm. Toxo IgG index of ≥1.00 or >8 IU/mL as prescribed by WHO for positive reaction. Samples with equivocal reaction having Toxo IgG index of 0.91 – 0.99 were retested.

**Serological testing of HIV antibody**: HIV testing was done according to the national serial algorithm using three rapid HIV Enzyme Immunoassay (EIA) kits – Determine HIV 1/2 (Abott, Japan), Unigold (Trinity Biotech, Ireland) and Stat-Pak HIV 1/2 (Chembio, USA). Determine HIV was used for first line testing followed by Unigold, while Statpak was used as a tie-breaker for samples with inconclusive results. All test protocols and interpretation of results were done according to the respective kit's manual.

**Clinical eye examination**: Eye examination on all consented participants included visual acuity using Snellen's chart/illiterate E chart at 6 meters. Anterior segment examination was done using pen torch; direct funduscopy was done using Beta 20 Heine (Germany) and dilated funduscopy where necessary to determine cases of ocular toxoplasmosis. Dilatation of pupil was done using 0.1% tropicamide. A diagnosis of ocular toxoplasmosis was made where there was the presence of a solitary inflammatory focus close to an old pigmented scar (satellite lesion), severe vitritis which may greatly impair visualization of the fundus although the inflammatory focus may still be discernable (headlight in the fog), retinal vasculitis, retinitis, old retinochoriodal scar, cystoid macular oedema and papillitis. Participants with such signs were identified, counseled and referred to University of Uyo Teaching Hospital for further management. Those clinically diagnosed with ocular toxoplasmosis were regarded as Presumed Ocular Toxoplasmosis (POT), while those with clinical signs and tested seropositive for anti-*T. gondii*IgG antibodies were regarded as sero-confirmed Ocular Toxoplasmosis (OT).

**Ethical considerations**: Ethical clearance was obtained from the Ethical Review Committee of the Akwa Ibom State Ministry of Health. Permission was obtained from the leaders of the respective livestock sellers and abattoir workers' association. Written informed consent was obtained from the participants before inclusion in the study.

**Statistical Analysis**: Data from the questionnaires, clinical eye examination, anti-*T.gondii*IgG antibody and HIV test results were analysed using the STATA statistical software version 12 (special edition) (STATA Corp, Texas 77845, USA). Chi Square was used to test for association between categorical variables and *P*<0.05 was termed significant.

## Results

There were 339 participants aged 15–78 (34.8±11.6) years, 283 were males and females 56, a total of 189 (55.8%) tested seropositive for anti-*T. gondii*IgG antibody. Factors associated with OT were age (31–50 years) and female gender (P = 0.049 and 0.001, respectively) (Table 1). Eight (2.4%) participants had POT; of this, 6(1.8%) were seropositive for anti-*T. gondii*IgG antibody, and 2 of the 8 POT (25%) were HIV-seropositive. Of the 189 persons having anti-*T. gondii*IgG antibody, 6(3.2%) had OT (Table 2). HIV infection was associated with POT (P=0.033) (Table 3). Most of the ocular lesions, 7(87.5%), were unilateral, affecting mostly the right eye, 6(75%), and located at the posterior pole and peripapillary area 7(77.7%) while 2 (22.2%) at peripheral retina (Table 4).

**Table 1:** Factors associated with ocular toxoplasmosis among study participants

Variables	Total N=339(%)	Presumed ocular toxoplasmosis (POT) (%)	P value	Sero-confirmed ocular toxoplasmosis (OT) (%)	P value
**Age group (year)**					
≤30	145 (42.8)	0 (0.0)	0.002+*	0 (0.0)	0.049+*
31–50	162 (47.8)	8 (100.0)		6 (100.0)	
Above 50	32 (9.4)	0 (0.0)		0 (0.0)	
**Sex**					
Male	283 (83.5)	3 (37.5)	0.004+*	1 (16.7)	0.001+*
Female	56 (16.5)	5 (62.5)		5 (83.3)	
**Occupation**					
Livestock sellers	166 (49.0)	3 (37.5)	0.184*	1 (16.7)	0.056*
Butchers/ meat	121 (35.7)	2 (25.0)		2 (33.3)	
sellers					
Middlemen	52 (15.3)	3 (37.5)		3 (50.0)	
**Drinking** **unpasteurized milk**					
Yes	151 (44.5)	2 (25.0)	0.307*	2 (33.3)	0.696*
No	188 (55.5)	6 (75.0)		4 (66.7)	
**Eating** **undercooked meat**					
Yes	103 (30.4)	2 (25.0)	1.00*	1 (16.7)	1.00*
No	236 (69.6)	6 (75.0)		5 (83.3)	

* Fischer's Exact Test

+ Significant p value

**Table 2:** Prevalence of Ocular Toxoplasmosis among those with and those without anti-*T. gondii*IgG antibody

Ocular toxoplasmosis	Presumed ocular toxoplasmosis among respondents (%)	Confirmed ocular toxoplasmosis among respondents (%)	Ocular toxoplasmosis among those seropositive for *T.* *gondii*IgG, n=189 (%)
Yes	8* (2.4)	6 (1.8)	6 (3.2)
No	331 (97.6)	333 (98.2)	183 (96.8)
Total	339	339	189

* blurring of vision (100 %)

**Table 3:** Frequency of HIV infection in participants with presumed and ocular toxoplasmosis

HIV status	No. tested	POT (%)	Non-POT (%)	P value	OT (%)	Non-OT (%)	P value
Infected	13	2 (15.4)	11 (84.6)	0.033+*	1 (7.7)	12 (92.3)	0.099*
Non- Infected	326	6 (1.8)	320 (98.2)		5 (1.5)	321 (98.5)	
Total	339	8 (2.4)	331 (97.6)		6 (1.8)	333(98.2)	

* Fischer's Exact Test

+ Significant p value

POT = Presumed ocular toxoplasmosis, OT = Ocular toxoplasmosis

**Table 4:** Distribution of lesions among eight respondents with POT/OT and their HIV status

Lesions	Frequency (%)	HIV seropositive
**Eye affectation**		
Right	6 (75)	0
Left	1 (12.5)	1
Both	1 (12.5)	1
**Location of the lesion in 9** **affected eyes**		
Papillomacula bundie	2 (22.2)	0
Macular	3 (33.3)	1
Peripapillary	2 (22.2)	1*
Peripheral	2 (22.2)	0

* One person with 2 affected eyes in the peripapillary site

## Discussion

*Toxoplasma gondii* infection is the most important cause of posterior uveitis, but the prevalence and incidence of ocular symptoms after infection largely depend on the socio-economic factors and the circulating parasite genotypes (16). In our study, the gender proportion of livestock farmers and slaughter house workers was in favour of males who dominated the workforce as documented in another study (17). Slaughter house work and livestock farming in this environment are mostly carried out by men, while the women may be involved as meat vendors. In this study, most of the workforce was aged 31–50 years (47.8%), and all the persons with either presumed ocular toxoplasmosis (POT) (n=8) or ocular toxoplasmosis (n=6) were aged 31–50 years which is the active workforce group in this environment (Table 1). Young persons' predominance observed in this study can be explained by the fact that this occupation requires exertion of physical energy which is abundant in them. Female gender was mostly affected by OT (62.5%) in this study (Table 1). This is important with respect to congenital toxoplasmosis and the fact that all of them were in the child bearing age (18).

Although there was no statistically significant association between occupation and POT/OT, middlemen who were directly involved in bulk purchase and retail sellers showed a near significant association (P=0.056). Possible causes of ocular manifestations of toxoplasmosis have not been fully elucidated, but it is believed that factors related to both the parasite and the host (genetic, age, immune status, inoculum) contribute to the development of this disease (19).

A total of eight (2.4%) persons were clinically diagnosed with presumed ocular toxoplasmosis (POT) in this study (Table 2). An earlier hospital-based study in a tertiary hospital in Uyo amongst patients attending an ophthalmology clinic reported POT prevalence to be 0.28% (11). Laboratory investigations in this study on POT cases revealed that six of the eight cases had anti *T. gondii*IgG antibody (Table 2). Of the two remaining POT cases that tested negative, one was with active lesion. From previous documentations, *T. gondii* specific IgG antibody does not appear until 1–2 weeks after infection (except IgM antibodies) and if present, the titre is usually very low and gradually builds up to reach the peak in 1–2 months (20). This study did not include IgM detection since it was centered on chronic exposure. Further explanation for the observed results could be based on the premise that if the IgG titers for toxoplasmosis are completely negative, down to a 1:1 dilution, then toxoplasmosis is completely ruled out in an immunecompetent person (21). However, an immune-suppressed patient could have a completely negative immunoglobulin titers and yet have active ocular toxoplasmosis (OT) infection (21). The two POT cases in this study were HIV seronegative. In cases like this where the diagnosis is uncertain, demonstration of anti-toxoplasma antibody titers and definitive diagnosis with Polymerase Chain Reaction (PCR) using aqueous and vitreous samples have been suggested (21).

Overall, the prevalence of OT among livestock farmers and raw meat handlers in Uyo was found to be 1.8%, while 3.2% was recorded for those that were seropositive for anti- *T. gondii*IgG antibody (Table2). A similar cross sectional population-based study in Ghana recorded a prevalence of 2.6% (22). However, a meta-analysis report in a tertiary hospital in Northern California (23) indicated that OT was diagnosed in 8.4% of patients with uveitis. The higher prevalence of OT noted in the Californian report could be due to the fact that it was a hospital-based study with bias for those with eye problems. Serological testing for *T. gondii*is is said to play little role in the diagnosis of ocular toxoplasmosis. Its primary use is in the exclusion of ocular toxoplasmosis as the cause of posterior uveitis if specific antibody is absent from the serum (24). Furthermore, due to the high seropositivity of *T. gondii* IgG in endemic areas, the positive predictive value of IgG is usually low, and a positive IgG cannot be interpreted as indicative of active toxoplasma infection (25). This study showed a high specific *T. gondii*IgG seroprevalence of 55.8% out of which 3.2% had OT. In a Brazilian study, 74·5% were seropositive for IgG anti-*T. gondii* antibody of which, 27·3% had O T (26). In Brazil, human infection is associated with an unusually high occurrence of ocular disease in some locations due to genotypic variations (27). There are different genotypes of *T. gondii* and the types that cause ocular complications differ from one region to another. The severity of ocular infection is largely determined by the immunity of the host, virulence of the parasites and environmental factors (28). The epidemiologic features suggest that infection with *T. gondii* in Brazil is more likely to lead to serious ocular disease, even in otherwise healthy persons (27). To the best of our knowledge, the genotype that causes ocular complications in Nigeria has not yet been determined and needs to be investigated. In this study, the most common ocular complain among the ocular toxoplasmosis cases was blurring of vision (100%) (Table 2) which was also reported in other studies (11). This study also revealed that most of the ocular lesions affected the posterior pole (77.7%) which explains the high percentage of blurring of vision complained by the patients.

The majority of immune-competent host ocular toxoplasmosis is often unilateral, whether occurring as primary or reactivated disease (29). In this study, most of the lesions were unilateral, only one was bilateral and this is consistent with findings in other studies (11,22). Two (25%) of the participants with POT in this study were HIV seropositive and there was a statistical significant association between HIV status and POT (Table 3). One of the HIV infected persons with bilateral lesions was undergoing anti-retroviral therapy suggesting subsistence of immunodeficiency as immuno-compromised persons are more prone to developing bilateral disease (24).

Of a total of nine eyes affected among the participants, 7(77.8%) of the lesions were located in the posterior pole, while 2(22.2%) were in peripheral retina (Table 4). This is similar to findings earlier reported (11,30). Holland *et al.* (30) suggested that the parasite preference for the posterior pole might reflect regional anatomical differences, including variations in the microvasculature and the distribution of immune cells. Seven of the nine lesions affected the right eye and the predominance of the right eye is probably because the right common carotid artery is the first branch of the aortic arch and so the haematological flow will more likely reach the right eye before the left (30).

The cow/goat sellers are very mobile people and difficult to gather in one place; we had to visit the slaughter houses several times to be able to track them down for blood sample. Further research on the molecular epidemiology of *Toxoplasma gondii*IgG antibodies amongst livestock farmers, butchers and meat sellers will be beneficial.

From the study, it was concluded that the prevalence of presumed ocular toxoplasmosis and ocular toxoplasmosis among livestock farmers and raw meat handlers in Uyo was significant (2.4% and 1.8% respectively). A significant proportion of these workers showed chronic exposure to *T gondii* infection (3.2%). The potential risk factors identified were being female gender, and those in the fourth and fifth decades of life, HIV seropositivity and occupational exposure, particularly those directly involved in bulk purchase and retail sellers. These findings are of immense public health concern, and it is recommended that the Ministry of Health in collaboration with the Ministry of Agriculture should step up health education and sensitization to the occupationally exposed groups.
